# Visual respiratory biofeedback to improve visuospatial cognition and cardiac interoception in migraineurs: a study protocol for a randomized controlled trial

**DOI:** 10.3389/fneur.2023.1197026

**Published:** 2023-07-05

**Authors:** Krithika A. Ramaswamy, Shivaprasad Shetty, Prashanth Shetty

**Affiliations:** ^1^Department of Yoga, SDM College of Naturopathy and Yogic Sciences, Ujire, Karnataka, India; ^2^SDM College of Naturopathy and Yogic Sciences, Ujire, Karnataka, India

**Keywords:** biofeedback, cognition, interoception, migraine, pranayama, yoga

## Abstract

**Objectives:**

Migraine is a complex neurological disorder that typically presents with unilateral cephalgia associated with cognitive impairment and reduced interoception. These symptoms result in socio-economic repercussions due to reduced productivity, efficiency, and work performance. Therefore, along with headache management, improving cognition and interoception should also be significant therapeutic targets to effectively manage migraine. To achieve this, we propose to explore the role of a yoga-based visual respiratory biofeedback (VRB) as a possible therapeutic strategy.

**Methods and analysis:**

At least 64 participants will be recruited for the trial after screening for eligibility criteria, using the migraine screening questionnaire and Montreal cognitive assessment test. They will be randomly allocated (1:1) to either the experimental group receiving a 20-min session of yoga-based VRB or the control group who will be asked to watch a documentary film for the same duration. Visuospatial cognition will be assessed by the Corsi block-tapping task, and cardiac interoceptive accuracy will be assessed by the heartbeat counting task at baseline and immediately after the intervention. Based on the distribution and variance of the data obtained, analysis will be conducted based on linear mixed models using SPSS version 28.0.1.0, with a two-sided *p*-value of < 0.05 considered to be statistically significant.

**Discussion:**

To the best of our knowledge, this is the first study to design and assess the effects of yoga-based biofeedback therapy on cognition and cardiac interoception in migraineurs. Furthermore, we postulated that pranayama's therapeutic effects might be enhanced by using visual yogic respiratory biofeedback. Considering the socio-economic burden of migraine, if found effective, VRB investigated in the trial could be considered as a therapeutic strategy.

**Clinical trial registration:**

ClinicalTrials.gov CTRI, CTRI/2023/03/050430.

## 1. Introduction

Migraine is a complex neurological condition that impacts a large proportion of the human population across all geographic locations and cultures. In the Global Burden of Disease studies initiated by the World Health Organization, migraine was first featured in the year 2000 as one of the contributing diseases. It ascended up the ladder from 19th in ranking in 2000 to 7th in 2010 (1), 6th in 2013 (2), and 7th in 2015. It is one among the eight chronic diseases affecting more than 10% of the population (3). Migraine affects one in 10 people worldwide and is more prevalent in women, students, and urban residents. A population-based study in Karnataka, Southern India, showed that the age-matched 1-year prevalence of migraine was 25.2%, greater in women and peaked between 35 and 45 years of age in both genders (4). The prevalence of migraine headaches among both adults and adolescent populations reflects a burdensome and debilitating condition that affects productivity and wellbeing (5).

Among other symptoms, patients with migraine are known to have a cognitive decline associated with structural brain changes (6), with migraine episodes frequently preceded by cognitive deficits (7). Being extremely common in the premonitory period of migraine, they are strong predictors of an attack (8). Cognitive impairments may linger as postdromes beyond the headache phase and may not be resolved by acute migraine medications (9, 10). Cognitive dysfunction not only presents during migraine attacks but also persists during the interictal period (11). The commonly reported symptoms during the interictal stage are executive or attentional dysfunction, impaired focus, and trouble thinking and reasoning. These symptoms can have significant socio-economic repercussions owing to absenteeism or poor work performance (12, 13).

Neuroimaging studies have consistently shown activation of the posterior cortical regions responsible for visuospatial cognition during all stages of migraine attacks (14). This points to selective impairment of the prefrontal and temporal cortices during migraine episodes. Migraine is also associated with dysfunction of the insula, the brain region implicated in cognition and interoception (15). Current conventional management for migraine focuses more on headache relief (16). The clinical symptoms of migraine-related cognition are key evidence of functional brain abnormalities underlying migraine pathogenesis. Additionally, the better interoceptive ability may help migraineurs in the early recognition of headache onset and also develop a more accurate awareness of triggering and aggravating factors. Hence, improvement of cognitive function and interoception should be valued as therapeutic targets as they may aid in improving the quality of life and minimizing loss of productivity in migraineurs.

The concept of interoception in modern neuroscience originated in the early 1900's (17). However, traditional Indian philosophies, such as yoga, have explored the significance of inward-directed attention for centuries. Yoga is a mind-body therapy and an ancient Indian science gaining popularity around the world. It includes various practices such as self-restraint (Yama), observance (Niyama), physical postures (Asana), breath regulation (Pranayama), withdrawal of the senses (Pratyahara), concentration (Dharana), meditation (Dhyana), and cleansing procedures (Kriya) (18, 19). Yogic concepts, such as self-awareness and mindfulness, have been extensively studied for their therapeutic benefits in various conditions including migraine (20–22). Previous studies have demonstrated that the practice of pranayama or yogic breath regulation significantly improved memory, perceptual sensitivity, focused attention, and cognitive functioning (23–26).

Pratyahara is a yogic state of interoception involving the withdrawal of attention from external distracting stimuli and directing the attention inward (27). According to the Ashtanga yoga of sage Patanjali, this state of mind arises after attaining mastery over the practice of pranayama or breath regulation (28). However, mastering the pranayama can be a daunting task and a time-consuming process for a beginner in yoga. Hence, while practicing pranayama, the use of visual respiratory biofeedback (VRB) may help hasten the learning process of the patient. At the same time, it also enables the treating yoga physician to objectively monitor the patient's breathing pattern.

Therefore, in this study, we propose the use of VRB as a therapy for improving cognition and interoception in migraineurs and to assist in the learning of yogic breathing or pranayama, thereby enhancing its therapeutic effect. We believe that completing this clinical trial would further add substantial evidence to the literature.

## 2. Methods

### 2.1. Study design and setting

This study was designed in conformance with Standard Protocol Items: Recommendations for Interventional Trials (SPIRIT) (29). It will be carried out as a single-center, open-label, randomized controlled trial. The trial is registered under CTRI (CTRI/2023/03/050430), and the institutional ethics committee approval has been obtained.

**Figure 1:**
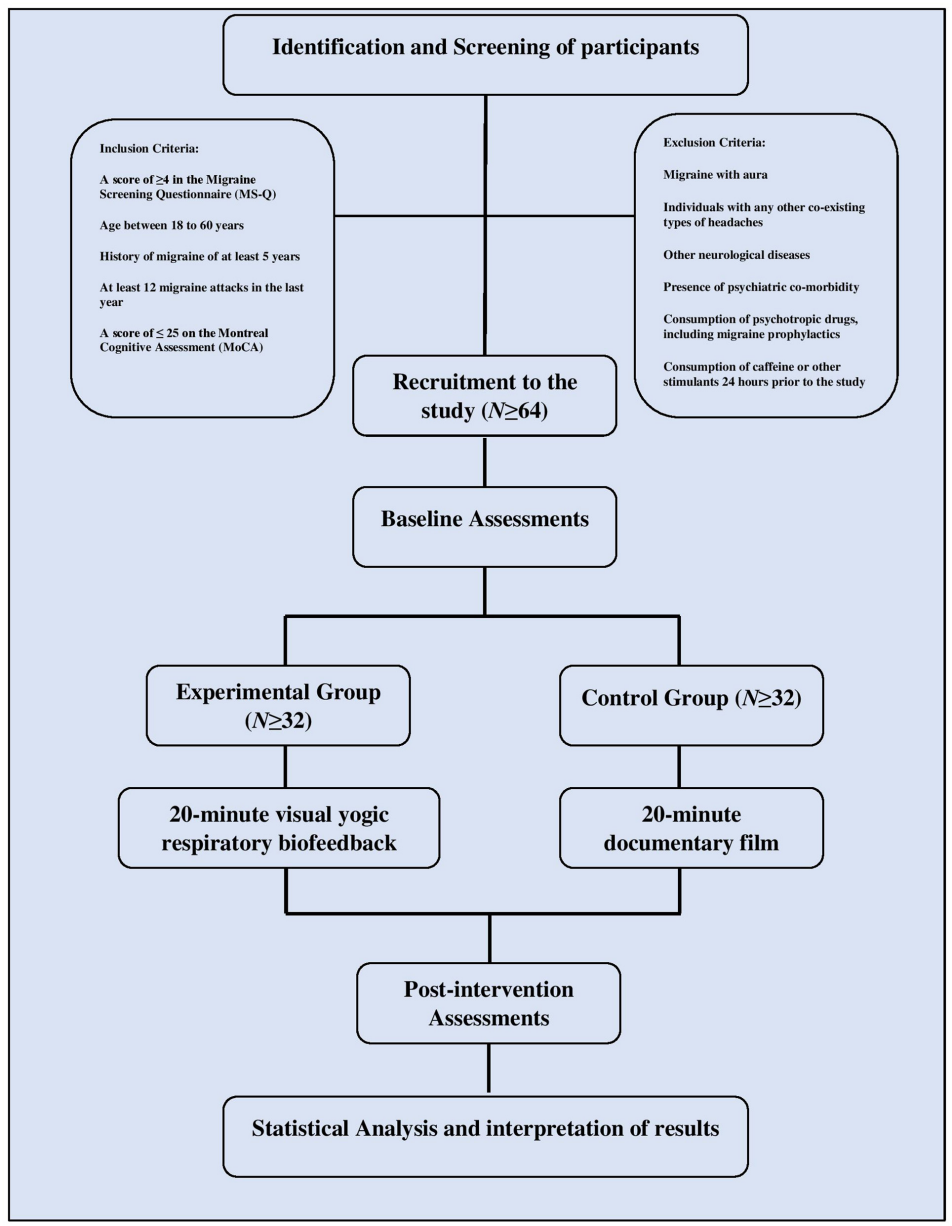
Trial flow diagram.

Participants will be recruited from SDM Yoga and Nature Cure Hospital, Dharmasthala, India, using advertisements on campus notice boards. Participants who come forward in response to advertisements will be given thorough verbal information about the study, and a written informed consent form to express their agreement to participate will be collected by the principal investigator. Before enrollment, participants will be screened for eligibility based on inclusion and exclusion criteria using a pre-designed proforma and will be administered the migraine screening questionnaire (MS-Q) and Montreal cognitive assessment test (MoCA) (30, 31). Those who meet the inclusion and exclusion criteria will be randomly allocated to either the experimental group or the control group (Figure 1). Based on the group assigned, participants will individually receive the intervention on the same day. Baseline and post-intervention assessments will be conducted within 15 min before and after the intervention. The assessment and intervention plan in this study are summarized in Table 1.

**Table 1:** Study protocol timeline.

**Timeline**	**Screening**	**Baseline**	**Intervention**	**Post-intervention**
Informed consent	•			
Demographic data	•			
MS-Q	•			
MoCA	•			
**Criteria**				
Clinical diagnosis based on the International Classification of Headache Disorders criteria	•			
Inclusion criteria	•			
Exclusion criteria	•			
**Intervention**				
Visual respiratory biofeedback			•	
Documentary viewing			•	
**Outcomes**				
Corsi block-tapping task		•		•
Cardiac interoceptive accuracy score		•		•

MS-Q, migraine screening questionnaire; MoCA, Montreal cognitive assessment test.

### 2.2. Inclusion criteria

Participants will be included in the study if they meet the following criteria:

- Migraine without aura diagnosed based on the International Classification of Headache Disorders (ICHD-3) criteria (32).
- Age between 18 and 60 years.
- A score of ≥4 on the migraine screening questionnaire (MS-Q).
- History of episodic migraine of at least 5 years.
- At least 12 migraine attacks in the last year.
- Participants in the interictal period.
- A score of ≤ 25 on the Montreal cognitive assessment (MoCA).

### 2.3. Exclusion criteria

Participants will be excluded from the study if they meet the following criteria:

- Migraine with aura.
- Chronic migraine.
- Any other co-existing types of headaches.
- Other neurological diseases.
- Presence of psychiatric co-morbidity.
- Consumption of psychotropic drugs, including migraine prophylactics.
- Consumption of caffeine or other stimulants 24 h before the study.

### 2.4. Sample size

The required sample size is estimated *a priori*. Based on previous literature assuming a medium-to-large effect size of 0.75 (51), a two-tailed, level 5% *t*-test requires a total of 29 patients per group to detect a respective group difference with a statistical power of 80%. Accounting for a potential loss of power because of a maximum of 10% dropouts, at least 64 participants will be recruited for the trial.

### 2.5. Randomization

Participants will be randomly allocated to the experimental group or the control group (1:1) by block randomization with randomly varying block lengths. The randomization list will be created by a colleague not involved in participant recruitment or assessment using Random Allocation Software (33). The list will be password-protected, and no person other than them will be able to access it. Based on this, sealed, sequentially numbered opaque envelopes containing the treatment assignments will be prepared. After obtaining written informed consent and baseline assessments, the study investigator will open the lowest numbered envelope to reveal that participant's assignment.

### 2.6. Study goals and objectives

The purpose of this study is to assess the impact of visual yogic respiratory biofeedback on visuospatial cognition in migraineurs without aura using the Corsi block-tapping task. In addition, this study will also examine its effects on cardiac interoceptive accuracy by the heartbeat counting task.

#### 2.6.1. Primary outcome

The primary outcome is the performance on the Corsi block-tapping task. The forward Corsi block-tapping task is a measure of visuospatial working memory. The electronic version of the test will be administered on Inquisit Lab software.

Participants are shown a screen with nine boxes. The boxes light up in a predetermined order which is consistent among participants. They are then instructed to click on the boxes in the same order they were illuminated. The sequence length begins at a level of two boxes and can be continued to level nine. Participants have two opportunities to attempt each length of the series. If one of the sequences was successfully entered, the next sequence begins. The Corsi span or length of the last correctly recalled sequence and the total number of correctly recalled sequences across the whole task are recorded. Then, the summary scores are obtained as described by Kessels et al. (34).

#### 2.6.2. Secondary outcome

The heartbeat counting task will be used to measure cardiac interoceptive accuracy. Participants will be instructed to place their right palm on the chest and concentrate on their own heartbeats and silently count them throughout four randomized intervals (lengths: 25, 35, 45, and 60 s), without receiving any information regarding the interval lengths or feedback on their performance quality. Participants will be encouraged to sit in a comfortable manner, prevent movements, and refrain from employing manipulative methods such as stopping breathing and taking their pulse. Importantly, they should only count heartbeats that they are certain of perceiving. An electronic start and stop signal will be delivered for each interval, following which participants must report their counted heartbeats. First, a 15-s training session will be performed, to familiarize them with the task.

The actual heartbeats will be recorded on the two-channel data acquisition system “Power Lab 15T” by AD instrument, Australia, acquired at a sampling rate of 1,024 Hz. The heart rate in beats per minute will be calculated by counting the R waves of the QRS complex in the ECG. The average heartbeat perception scores will be calculated using the equation below. The score ranges from 0 to 1; higher scores indicate a higher IAc for cardiovascular signals.


$$\begin{array}{l} IAc\;Score\;=\;\\ \frac{1}{4}\sum (1-\frac{\left(\left|recorded\;heartbeats-counted\;heartbeats\right|\right)}{recorded\;heartbeats}) \end{array}$$

### 2.7. Intervention

The experimental group will be administered a 20-min yogic VRB session. The protocol for the biofeedback will be designed *a priori* (35). Participants in the control group will be asked to watch a documentary film for a duration of 20 min. The interventions are described in conformance with the TIDieR guidelines (36).

#### 2.7.1. Yogic VRB

The intervention will take place in a dimly lit, sound-attenuated room. Participants will be asked to sit on an armless chair with back support by placing their feet on a non-conducting material. A 39.62 cm screen will be placed 1 m away from the chair at the eye level of the participant, on which VRB will be presented.

The participants' respiration will be assessed by attaching a respiratory effort transducer about 8 cm above the lower coastal margin. Care will be taken to tighten the transducer in such a way that full inspiration is not hindered. The transducer will be connected to a two-channel data acquisition system “Power Lab 15T” by AD instrument, Australia. The respiratory waveform will be presented on the screen as visual biofeedback.

Participants will be instructed to perform alternate nostril yoga breathing (ANYB or *nadishuddhi pranayama*) at the rate of 6 breaths/min with an emphasis on deep abdominal breathing. The technique of ANYB entails alternating breathing through the left and right nostrils. The thumb and ring fingers of the right hand are employed to manipulate or occlude the nostrils in this nostril manipulation pranayama. Throughout this exercise, the focus is on the breath and breathing. Participants will be asked to use the visual biofeedback to guide their rate and depth of breathing.

Instructions for pranayama practice will be given by an experienced yoga physician. The physician will also continually monitor the breathing pattern of the participant and encourage them to adhere to the breathing instructions. A 2-min training session will be conducted before the actual 20-min intervention for the participants to familiarize themselves with the procedure.

#### 2.7.2. Documentary film viewing

Participants in the control group will be seated on the same chair as those in the experimental group, and the arrangements of the screen distance, height, and screen resolution will be the same as that of the experimental group to reduce environmental bias. The content of the documentary film will be unrelated to yoga and will not have emotionally stimulating material to have a neutral visual stimulus.

### 2.8. Data management and statistical analysis plan

Primary data acquisition will be performed by the principal investigator. It will be entered into an electronic data capturing (EDC) system and then formatted into a tabular form for analysis. Each subject will be assigned a unique code to mask their identity.

Data will be analyzed using SPSS version 28.0.1.0 [Computer software]. The distribution and variance of data will be analyzed by testing for normality. The analysis will be based on linear mixed models. Continuous variables with normal distributions will be expressed as means and standard deviations, while those with skewed distributions will be expressed as median and interquartile ranges. A two-sided *p-value of* < 0.05 will be considered statistically significant.

### 2.9. Safety considerations

To the best of our knowledge, there has only been a single reported case study of spontaneous pneumothorax induced by a fast yogic breathing technique, *kapalabhati*. However, in contrast to this advanced yogic breath technique, ANYB, which the current study proposes to use, is a slow and simple breathing technique. The intervention will be administered by an experienced yoga physician inside a hospital equipped to provide an emergency medical response.

During the course of the study, if any participant reports an adverse event, the intervention will be immediately terminated and appropriate medical care will be given. For every enrolled participant, any adverse events will be noted down in a log book which will be periodically submitted to the Institutional Ethics Committee.

## 3. Discussion

To the best of our knowledge, this is the first study to design and evaluate the effects of yoga-based biofeedback therapy on cognition and cardiac interoception in migraineurs. Additionally, we proposed that the use of yogic VRB may enhance the therapeutic effects of pranayama.

There are no previous studies on yogic biofeedback, although many studies support the use of physiological biofeedback in the management of migraine. A meta-analysis on biofeedback for migraine revealed that it is a non-pharmacological pain management strategy, which is as effective as pharmacological medication (37). Migraine patients typically exhibit a changed set point of the autonomic nervous system with a tendency for sympathetic activation (38). Biofeedback training is thought to improve autonomous nervous system activity and make migraineurs more resilient to triggers that might cause headaches (39). Biofeedback therapies that target pathophysiological pathways in migraine have consistently shown better clinical efficiency (40). Biofeedback has also been shown to improve interoception as evidenced by a study that found visual biofeedback led to improved sensitivity to internal cardiac signals and significantly higher high-frequency power (HF) in HRV probably as a result of cardiac autonomic modulation (41).

This study proposes to exclude patients with migraine with aura and include only patients with migraine without aura, though both do overlap in their epidemiology and clinical presentation. However, evidence suggests that the two entities inherently differ in pathophysiological mechanisms with imaging studies showing differences in brain activation patterns between the two in the interictal phase, which may probably be due to hyperresponsiveness of the visual cortex in migraine with aura.

This study is strengthened by the methodological rigor added by the randomized controlled trial design. Since the study also requires only a single lab visit by the participant, fewer dropouts are expected. The trial protocol is reported using SPIRIT and TIDieR guidelines making the study methods and analysis more transparent and enhancing the relevance of the trial and the results.

One of the main limitations of the current study is a lack of follow-up testing to evaluate the delayed effects of the intervention. Owing to the nature of the yoga-based intervention, blinding of the participants was not possible. Further studies with more robust designs can attempt to address these shortcomings.

Given the substantial loss in productivity and efficiency due to migraine-related cognitive impairment, the results from this study could have good translational significance. If found effective, respiratory biofeedback would enhance the therapeutic effects of yoga breathing or pranayama in improving cognition and interoception. This could be explored as a short-duration, non-invasive strategy in managing migraine, which would also improve quality of life.

## Ethics statement

The studies involving human participants were reviewed and approved by Institutional Ethics Committee, SDM College of Naturopathy and Yogic Sciences and CTRI (CTRI/2023/03/050430). The patients/participants provided their written informed consent to participate in this study.

## Author contributions

KR and SS contributed to the conception and design of the study and wrote the first draft of the manuscript. PS wrote sections of the manuscript. All authors contributed to the manuscript revision, read, and approved the submitted version.
